# Estimation of Human Workload from the Auditory Steady-State Response Recorded via a Wearable Electroencephalography System during Walking

**DOI:** 10.3389/fnhum.2017.00314

**Published:** 2017-06-13

**Authors:** Yusuke Yokota, Shingo Tanaka, Akihiro Miyamoto, Yasushi Naruse

**Affiliations:** ^1^Center for Information and Neural Networks (CiNet), National Institute of Information and Communications Technology, Osaka UniversityKobe, Japan; ^2^Sawamura Prosthetics and Orthotics Service Co., Ltd.Kobe, Japan

**Keywords:** workload, electroencephalogram, auditory steady-state response (ASSR), n-back task, real world recording

## Abstract

Workload in the human brain can be a useful marker of internal brain state. However, due to technical limitations, previous workload studies have been unable to record brain activity via conventional electroencephalography (EEG) and magnetoencephalography (MEG) devices in mobile participants. In this study, we used a wearable EEG system to estimate workload while participants walked in a naturalistic environment. Specifically, we used the auditory steady-state response (ASSR) which is an oscillatory brain activity evoked by repetitive auditory stimuli, as an estimation index of workload. Participants performed three types of N-back tasks, which were expected to command different workloads, while walking at a constant speed. We used a binaural 500 Hz pure tone with amplitude modulation at 40 Hz to evoke the ASSR. We found that the phase-locking index (PLI) of ASSR activity was significantly correlated with the degree of task difficulty, even for EEG data from few electrodes. Thus, ASSR appears to be an effective indicator of workload during walking in an ecologically valid environment.

## Introduction

The workload of the human brain changes constantly throughout the day according to internal brain state. The workload is high for active states, such as thinking and concentrating, and low in resting states. The ability to estimate workload may enable the evaluation of internal brain state. This could have a number of practical applications, such as in systems that measure the fatigue level of drivers during the operation of a vehicle, evaluations of the mental load of exercise during rehabilitation in hospitalized patients, and assessments of the mental load of pilots during operation of an airplane.

In this study, we used the auditory steady-state response (ASSR) to estimate brain workload. The ASSR is an oscillatory brain signal evoked by repetitive auditory stimuli. In general, the ASSR is known to be most strongly evoked by a 40 Hz auditory stimulus (Galambos et al., 1981; Ross et al., 2000). Recent studies have shown that the 40 Hz ASSR changes according to the difficulty of a corresponding task (Tiitinen et al., 1993; Griskova et al., 2007, 2009; Griskova-Bulanova et al., 2011; Roth et al., 2013; Yokota and Naruse, 2015). For example, Griskova-Bulanova et al. (2011) observed attenuated phase synchronization of the 40-Hz ASSR in a visual search task. They suggested that the workload related with visual modality has induced desynchronization of 40 Hz ASSR. Previously, we observed a change in the ASSR associated with cognitive load (Yokota and Naruse, 2015). Thus, it appears that the ASSR can be modulated by workload changes in several modalities. Frequently, the goal of previous studies has been to precisely characterize the ASSR, and so participants have been encouraged to remain motionless to reduce instances of movement artifact. Moreover, many previous studies used conventional electroencephalography (EEG) or magnetoencephalography (MEG) devices that are not suitable (i.e., too large) for measuring brain activity in ecologically valid environments. Thus, such studies have not thoroughly examined neural activity during activities of daily living, such as walking in a naturalistic environment. To address this, we sought to develop a novel method for estimating the workload of brain activity recorded in an ecologically valid environment.

Several studies have attempted to investigate neural activity during walking (Bulea et al., 2014; Ehinger et al., 2014; Kline et al., 2014; Lau et al., 2014; Lin et al., 2014; Seeber et al., 2014, 2015; Wagner et al., 2014). Some studies have reported that complex denoising methods are not necessary to extract brain activities during walking. Nathan and Contreras-Vidal (2016) suggested that the motion artifacts in slower walking rarely contaminate the EEG data. Moreover, Debener et al. (2012) and De Vos et al. (2014) were able to detect the P300 caused by odd-ball tasks without complex denoising methods during walking. However, artifacts such as muscle potentials and oscillatory noises caused by walking can contaminate EEG data. Therefore, to increase the signal to noise ratio, many studies have employed spatial filters, such as independent component analysis (ICA), to separate the EEG signal from the artifact (Lau et al., 2012, 2014; Bulea et al., 2014; Kline et al., 2014; Reis et al., 2014; Wagner et al., 2014).

Successful application of a spatial filter to an EEG signal requires data from many channels, thus necessitating EEG devices that can simultaneously record from more than 19 channels. In past studies that measured ERP during walking, the researchers removed the noise by using spatial filters such as ICA. In this study, we tried to reduce artifacts in the time domain rather than in the spatial domain. Kline et al. (2015) reported that the frequency of noise caused by walking was mainly lower than the gamma frequency band even if participants walked at faster speeds. Therefore, we focused on the high frequency band (around 40 Hz). Because the ASSR is known to be strongly evoked at 40 Hz (Galambos et al., 1981; Ross et al., 2000), it is possible to extract the ASSR modulation caused by the workload from the EEG data in which noise caused by walking is contaminated, without any pre-processing such as ICA. The large size and weight of such conventional EEG devices limits the use of these technologies in naturalistic settings. Moreover, the time required to place numerous EEG electrodes on the head of each participant further reduces the accessibility of this approach.

In this study, we developed a novel method for estimating brain workload from a small number of data channels recorded via a portable EEG system worn while walking in a naturalistic environment. The wearable EEG system consisted of a portable EEG device (Weight: 80 g, Size: W52 mm × D50 × H20 mm) that could be attached to custom-designed headgear. We used seven electrodes to measure EEG, and placed a single electrode at the anterior, three electrodes at the front-central and three electrodes at the occipital location. Because we used active electrodes, we were able to measure highly reliable data using dry electrodes without conductive paste (Higashi et al., in press). As in our previous studies, we used the ASSR to estimate workload and employed an N-back task to regulate the degree of task difficulty. We hypothesized that the ASSR changes according to the difficulty of a corresponding task.

## Materials and Methods

### Participants

Fifteen participants (male 7, female 8; age range 20–35 years) took part in this study. All participants had normal hearing and normal or corrected-to-normal vision. Participants provided informed written consent after the details of the procedure had been explained. All experimental procedures were approved by the Ethical Committee for Human and Animal Research of National Institute of Information and Communications Technology. All experiments were performed in accordance with the ethical standards described in the Declaration of Helsinki.

### Experimental Procedure

**Figure 1:**
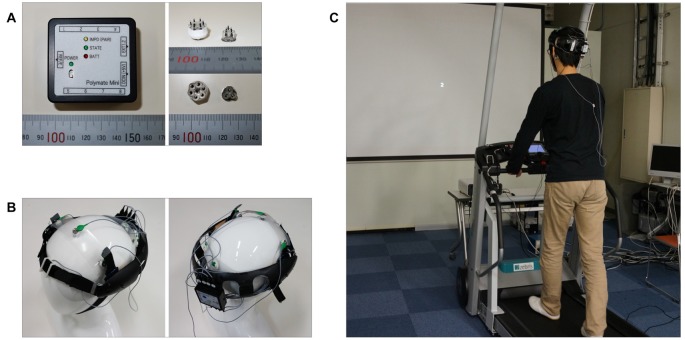
Wearable electroencephalography (EEG) system and experimental setup. **(A)** Portable EEG device (Polymate Mini AP108) and dry electrodes. **(B)** Custom-designed headgear. **(C)** Experimental setup.

Schematic illustrations of the experimental setup are shown in Figure 1. Participants were asked to perform the N-back task while walking on a treadmill. The N-back task is a continuous performance task that has been used to study short-term working memory (Owen et al., 2005). The experimental stimuli included the visual stimuli in the N-back task and the repetitive auditory stimuli used to evoke the ASSR. Visual stimulus presentation was controlled via Visage (Cambridge Research System, Rochester, UK) and presented on a screen using an LCD projector (Canon, WUX400ST, Japan). Participants viewed the screen while walking on a treadmill (Zebris, FDM-T, DEU) at a speed of 2 km/h (Figure 1C). The walking speed was set to be equivalent among all trials to ensure that the noise caused by walking was similar among all the tasks. Several previous studies that recorded EEG during walking set the walking speed at approximately 2 km/h (Seeber et al., 2014, 2015; Wagner et al., 2014). Therefore, we adopted a walking speed of 2 km/h in this study.

**Figure 2:**
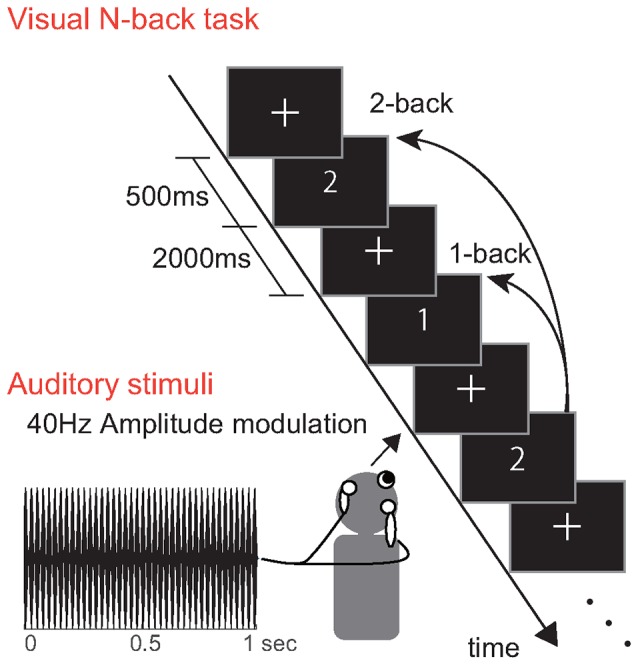
Experimental procedure. Participants performed an N-back task with visual stimuli “1” and “2”. The auditory stimuli had a carrier frequency of 500 Hz and 40 Hz amplitude modulation.

Visual stimuli were presented to the participants during the N-back task. A schematic illustration of the visual stimulus presentation is shown in Figure 2. In all experiments, we randomly presented either the characters “1” or “2” (each at a rate of 50%) on the screen for 500 ms. Each task comprised 70 trials, and a blank image with a fixation point was presented for 2000 ms between trials. One block included three different tasks: no-load (NL), 1-back and the 2-back task. In the NL task, participants performed visual discrimination. Specifically, they reported the current number shown on the screen by pressing a corresponding button. In the 1- and 2-back tasks, participants reported whether the current number matched the one from the previous N numbers. Participants used the index or middle finger of their right hand to press the buttons. The tasks in the first block were performed in the following order: NL, 1-back and 2-back tasks. The tasks in the second block were performed in the following order: 2-back, 1-back and NL tasks. All participants performed a practice test for approximately 10 min before beginning the experiment.

The auditory repetitive stimuli consisted of a 500 Hz pure tone with amplitude modulation at 40 Hz. The stimuli were transmitted to the participant via canal type earphones (Elecom, EHP-CH3000, JPN). The depth of the amplitude modulation of the sounds was 100%. The average A-weighted sound pressure level of the auditory stimulus was 63 dB for both ears. The participants were instructed to ignore the auditory stimuli, which were constantly presented to them while they performed the tasks.

We measured EEG responses using an 8-channel wireless EEG system (Miyuki Giken, Polymate Mini AP108 (W52-D50-H20 mm, 80 g), Japan) with active dry electrodes (Unique Medical, unique development, Japan) positioned at the following sites: Fpz, FC3, FCz, FC4, O1, Oz and O2. The participants wore custom-designed headgear (Sawamura Prosthetics and Orthotics Service, unique development, Japan) with the attached electrodes and wireless EEG device. EEG data were sampled at 500 Hz. All recorded signals were referenced to the left mastoid. The ground electrode was placed on the right mastoid.

### EEG Data Analyses

**Figure 3:**
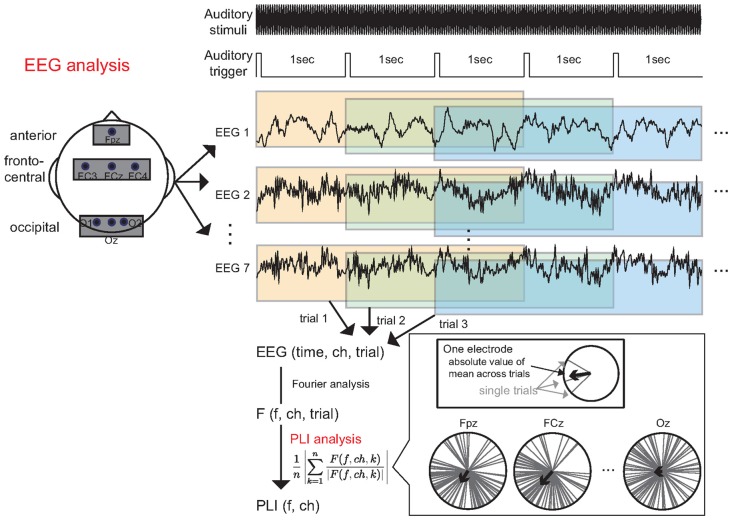
Data analysis. The continuous EEG data were divided into 3000 ms epochs based on triggers that occurred every second. Subsequently, each epoch was subjected to Fourier analysis to calculate the frequency response. Finally, we calculated the 40 Hz phase-locking index (PLI) from the frequency response.

A schematic illustration of the data analysis is shown in Figure 3. EEG analyses were performed using MATLAB (MathWorks, Inc., Natick, MA, USA). For each channel, the EEG data were divided into 3000 ms epochs on the basis of the auditory trigger, which occurred every second in the continuous EEG data. Thus, the auditory trigger rose every time that the amplitude modulation of auditory stimuli occurred 40 times. We discarded the EEG epochs of the first five trials for each task, to investigate brain activities after steady-state condition was attained under the auditory stimuli condition. We did not perform any pre-processing to remove artifacts caused by eye movement. We performed Fourier analysis for each epoch to calculate the frequency response.

Previous studies have suggested that the phase-locking index (PLI) of an ASSR can be modulated by changes in workload (Griskova-Bulanova et al., 2011; Yokota and Naruse, 2015). Thus, we focused on the PLI of the ASSR. The PLI was defined by the following formula:

(1) $$PLI=\frac{1}{n}\left|\sum_{k\text{ = 1}}^{n}\frac{F\left(f\text{, }ch\text{, }k\right)}{\left|F\left(f\text{, }ch\text{, }k\right)\right|}\right|$$

where *F* is the Fourier response, *f* is the frequency, *ch* is the channel number, *k* is the trial number and *n* is the number of trials. We used the average value of two PLIs obtained in two experimental blocks as an index of workload. We calculated the PLI for each participant at each electrode, and then averaged the PLI within each electrode location: anterior (Fpz), fronto-central (FC3, FCz and FC4), and occipital (O1, Oz and O2). We combined data from three electrodes in the fronto-central and occipital regions to make the data more robust with respect to environmental noise. Finally, we calculated the grand-averaged PLI using the average PLI of each electrode location in all participants. A schematic illustration of the three locations is shown in Figure 3. Data from our previous MEG study (Yokota et al., 2015) indicated that the ASSR data collected at the occipital electrode is important for estimating the workload, even though the ASSR at this location is relatively small. Thus, we sought to observe ASSR modulation via the occipital electrodes.

### Statistical Analyses

We performed the Lilliefors test for all data to validate whether the data were normally distributed. To validate the sphericity, we performed Mendoza’s multisample sphericity test for all data. For the data in which the sphericity was not validated, we corrected *p* values using the Greenhouse-Geisser method.

For the behavioral data (reaction time data and accuracy data), we used a repeated measures analysis of variance (ANOVA) for data in which the normality was validated, and we used the Friedman test for data in which the normality was not validated. For the behavioral data, the independent variable was task difficulty.

For the PLIs, we performed the Rayleigh test for each PLI value to verify whether the PLIs were statistically significant and then performed an ANOVA. The independent variables in the ANOVA were task difficulty, electrode location and block (experimental order).

For *post hoc* test (multiple comparison), we used *t*-tests or the Wilcoxon signed-rank test. The *p* values were corrected by the Shaffer method.

## Results

### Behavioral Data

**Figure 4:**
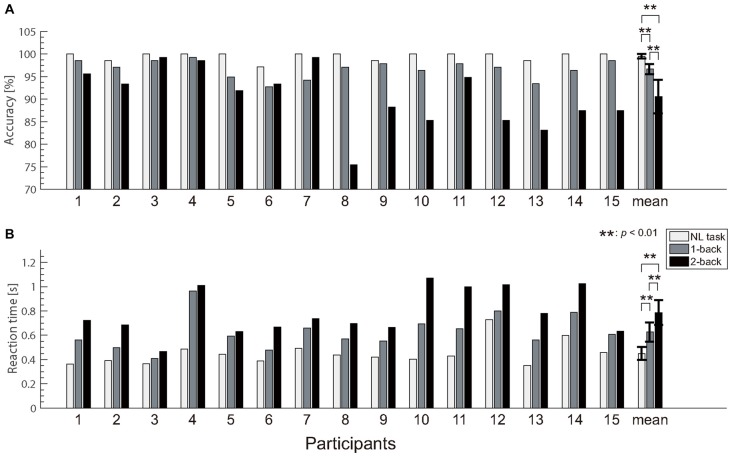
Behavioral results. **(A)** Accuracy for button pressing during each task. **(B)** Reaction time. We observed significant differences in both behavioral measures between the no-load (NL) and N-back tasks. Error bars indicate the 95% confidence limits of the mean.

The accuracy and reaction time data for the N-back tasks are shown in Figure 4. Because the normality was not rejected for the reaction time data (*p* = 0.173), we performed a repeated measures ANOVA for reaction time. In contrast, because the normality was rejected for the accuracy data (*p* < 0.001), we performed the Friedman test for the accuracy. A one-way repeated measures ANOVA revealed significant differences in the reaction time (*F*_(1.5,14)_ = 53.7; *p* < 0.0001; $\eta_{\text{p}}^{2}$ = 0.793) between the different tasks. Subsequent multiple comparison tests also revealed a significant difference in the reaction times for different tasks, such that the NL task was associated with a faster reaction time than the 1- and 2-back tasks (*t*_(14)_ = 6.50; *p* < 0.0001, *t*_(14)_ = 8.25; *p* < 0.0001), and the 1-back task was associated with a faster reaction time than the 2-back task (*t*_(14)_ = 5.80; *p* < 0.0001). The Friedman test revealed significant differences in the accuracy (*χ*^2^ = 25.2; *p* < 0.0001; *η*^2^ = 0.84). Subsequent multiple comparison tests revealed a significant difference in the accuracy for the different tasks, such that the NL task was associated with greater accuracy than the 1- and 2-back tasks (*Z* = 3.41; *p* < 0.0001, *Z* = 3.41; *p* < 0.0001), and the 1-back task was associated with greater accuracy than the 2-back tasks (*Z* = 2.78; *p* < 0.01). These results indicate that the NL and N-back tasks successfully varied with the task difficulty. The reaction times for all participants increased according to the task difficulty. Additionally, task accuracy decreased as the task difficulty increased in 12 participants.

### EEG Data

**Figure 5:**
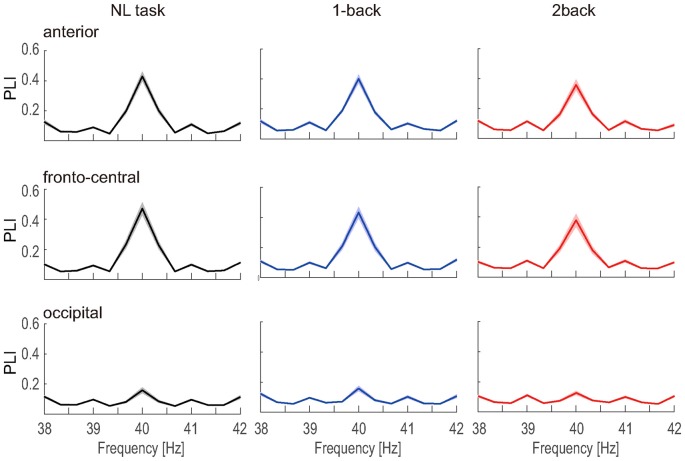
PLI vs. frequency. The grand-averaged PLI vs. frequency measured at the anterior, fronto-central and occipital electrodes. The shaded areas indicate the standard error.

**Figure 6:**
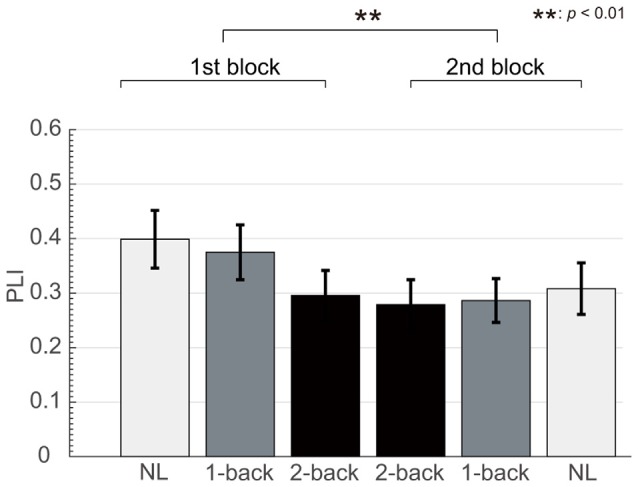
Change in fronto-central PLI according to experimental block. We observed significant differences in the auditory steady-state response (ASSR) between the first and second block.

**Figure 7:**
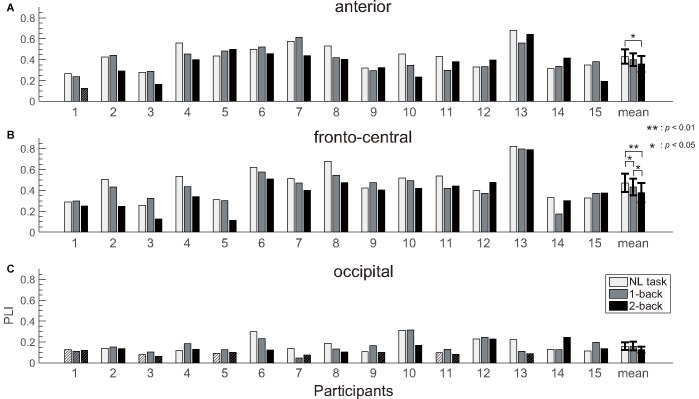
PLI results. The PLIs measured at the **(A)** anterior, **(B)** fronto-central and **(C)** occipital electrodes of the participants and the grand averaged value. We observed significant differences in the ASSR between the NL and 2-back task at the anterior, and between each tasks at the fronto-central electrodes. Error bars indicate the 95% confidence limits of the mean. The hatched bars indicate the PLI value that was below a threshold value of random variations tested using the Rayleigh test.

The number of epochs in the PLI analysis for each participant is shown in Table 1. The number of epochs was not entirely consistent between participants because we experienced occasional and transient loss of the wireless connection. The grand-averaged PLIs for each frequency for the three electrode locations are shown in Figure 5. We observed clear peaks in the 40 Hz PLI at the anterior and fronto-central electrodes. The grand-averaged PLI values among all the electrodes over all the participants according to the experimental blocks are shown in Figure 6. The 40 Hz PLIs for the three electrode locations (anterior, fronto-central and occipital) for each participant are shown in Figure 7. The PLIs were averaged over blocks. The hatched bars indicate PLIs that were not statistically significant at the five percent level.

**Table 1:** The number of epochs in the phase-locking index (PLI) analysis for each participant.

Task	Block	1	2	3	4	5	6	7	8	9	10	11	12	13	14	15
NL	1	92	118	138	137	107	141	96	148	138	135	131	146	148	141	140
	2	82	127	142	110	141	134	139	141	131	120	130	141	152	143	141
1-back	1	57	123	140	96	140	140	140	144	130	133	128	145	145	194	145
	2	98	132	147	142	133	142	136	141	126	113	146	144	149	144	140
2-back	1	80	133	138	139	139	137	141	143	132	139	131	140	148	145	143
	2	92	116	129	137	141	132	139	143	126	132	138	144	146	142	139

The Rayleigh test revealed that more than one-third of the occipital PLIs (13/45) were lower than significant value. This result suggests that it is difficult to capture activities related to ASSR at the occipital electrode location. Thus, we removed the occipital data for the following analysis. Because the normality was not rejected for the anterior and fronto-central PLIs (*p* = 0.518, *p* = 0.802), we performed a repeated measures ANOVA for PLI. We performed a three-way ANOVA with task difficulty (NL, 1- and 2-back task), electrode location (anterior and fronto-central) and block (first block and second block) as independent variables. A three-way repeated measures ANOVA of the PLI revealed the main effects of task difficulty (*F*_(1.67,14)_ = 9.49; *p* < 0.01; $\eta_{\text{p}}^{2}$ = 0.404) and block (*F*_(1,14)_ = 13.5; *p* < 0.01; $\eta_{\text{p}}^{2}$ = 0.491). We observed a significant interaction between task difficulty and block (*F*_(1.94,14)_ = 5.92; *p* < 0.01; $\eta_{\text{p}}^{2}$ = 0.298). Subsequent multiple comparison tests revealed significant effects of the task, such that the NL task elicited a higher PLI than the 1-back and 2-back task (*t*_(14)_ = 2.37; *p* < 0.05, *t*_(14)_ = 4.08; *p* < 0.01), and the 1-back task elicited a higher PLI than the 2-back task (*t*_(14)_ = 2.23; *p* < 0.05). We did not observe any significant interaction between task difficulty and electrode locations. However, in order to investigate which location showed the clearest change according to the task difficulty, we performed a multiple comparisons analysis. Subsequent multiple comparisons tests revealed significant effects of task difficulty at the anterior electrode, where the NL task elicited higher PLIs compared with the 2-back task (*t*_(14)_ = 2.87; *p* < 0.05). Likewise, multiple comparison tests revealed significant effects of task difficulty at the fronto-central electrodes, where the NL and 1-back task elicited higher PLIs compared with the 2-back task (*t*_(14)_ = 3.75; *p* < 0.01, *t*_(14)_ = 2.20; *p* < 0.05), and the NL task elicited higher PLIs compared with the 1-back task (*t*_(14)_ = 2.21; *p* < 0.05). The individual data showed that the PLIs elicited by the 2-back task at the fronto-central electrodes were smaller than those elicited by the NL-task, which was less difficult, in 13 of 15 participants.

**Figure 8:**
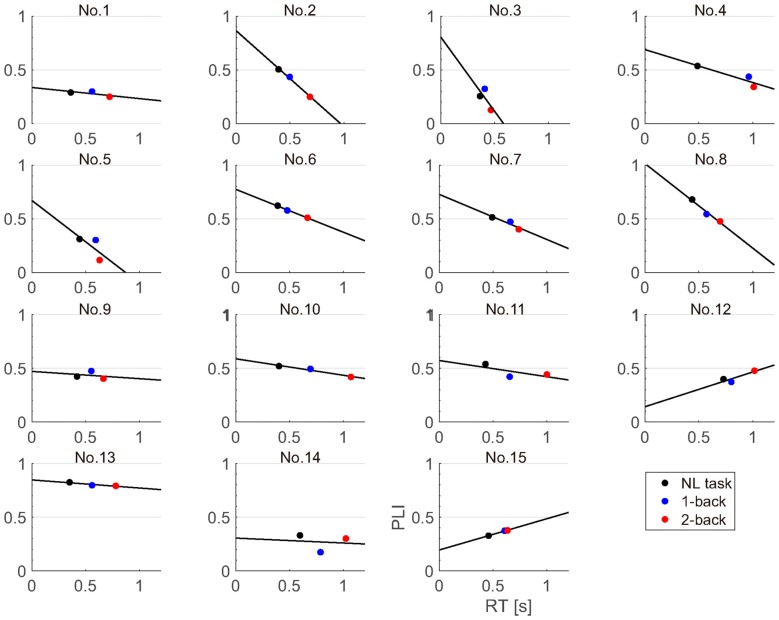
Correlation of reaction times and fronto-central PLIs for each participant.

Because the fronto-central PLI was most significantly modulated by task difficulty, we investigated the relationship between the fronto-central PLI and reaction time. We performed a regression analysis for the fronto-central PLIs and reaction times for each participant (Figure 8). We found that the regression coefficient was significantly negative (sign test; *p* < 0.01).

## Discussion

In the present study, we developed a wearable EEG system and were able to use the PLI of the ASSR to estimate the workload associated with NL and N-back tasks while participants wearing the portable EEG system walked in an ecologically valid environment. As in our previous study, the reaction time and accuracy in the NL and N-back tasks were modulated according to task difficulty. We also found that the reaction times of all participants increased according to the task difficulty. The accuracies of the NL task were higher than those of the N-back task for all participants. These results indicate that the N-back tasks successfully modulated the participant workload. The PLI of the ASSR measured by the wearable EEG system significantly decreased as the task difficulty increased. This result is consistent with our previous MEG study (Yokota and Naruse, 2015). Moreover, the PLI was modulated not only according to task difficulty but also the block (experimental order). This result suggests the possibility that the PLI was modulated according to the fatigue level of the participants as they performed the experiments. As reflected in the PLI, ASSRs were strongly evoked at the anterior and fronto-central electrodes. The PLI values at the fronto-central electrodes decreased according to the increase in reaction time. This result indicates that the PLI decreases according to increases in the task load. The PLI is the index measuring the amount of synchronization between the ASSR and the auditory stimuli. Therefore, the decrease in the PLI within the anterior electrode or fronto-central electrodes according to the task load reflects a decrease in the synchronization between the ASSR and auditory stimuli.

The PLI values at the fronto-central electrodes and anterior electrode can be used for workload estimation while the participant is walking. Moreover, the individual data showed that, among the different tasks, the fronto-central PLIs elicited by the 2-back task were the smallest in most of the participants. This result demonstrates the robustness of our method. In this study, we employed three EEG channels in the fronto-central electrode location and one at the anterior electrode location. Thus, it appears to be possible to estimate workload from EEG data recorded with few channels.

In this study, we found a maximum PLI at the fronto-central electrodes. Previous studies have reported a larger 40 Hz ASSR over the frontal area (Tiitinen et al., 1993; Griskova et al., 2007, 2009; Krishnan et al., 2009; de Jong et al., 2010; Griskova-Bulanova et al., 2011; Roth et al., 2013). Many studies have suggested that the ASSR source is mainly localized in the primary auditory cortex (Kuriki et al., 1995; Pantev et al., 1996; Gutschalk et al., 1999; Engelien et al., 2000; Herdman et al., 2002; Weisz et al., 2004). Thus, the maximal value of the ASSR observed at the fronto-central electrodes is considered to be due to the contribution of the primary auditory cortex. The occipital electrodes are far from the primary auditory cortex. Thus, the PLIs at the occipital electrodes were small since it is difficult to record the ASSR with the occipital electrodes.

In this study, all participants walked at a constant speed. Thus, we were able to control the amount of noise caused by walking among participants. The walking speed that we used in this study was close to the average walking speeds used in some previous studies (Seeber et al., 2014, 2015; Wagner et al., 2014). However, there is a possibility that some participants felt uncomfortable at the prescribed walking speed. Participants’ feeling of discomfort, may indicate increased workload caused by the walking speed. It is possible that this is a factor in the variation in PLI between participants.

Several previous studies have successfully used ASSR to define a relationship between workload and task difficulty (Griskova et al., 2007, 2009; Griskova-Bulanova et al., 2011; Roth et al., 2013; Yokota and Naruse, 2015). However, although many studies on EEG measurement during walking have been published, to the best of our knowledge, ours is the first study to estimate workload from the ASSR while participants were walking. Moreover, our wearable EEG system does not require conductive paste, thus reducing the time required to position the electrodes on the heads of the participants. As a result, we believe our system is superior in terms of application and ease of use. Our analysis method for ASSR did not include a spatial filter, which is commonly used for noise reduction for data collected while a participant is walking. Instead, we performed Fourier analysis to obtain the phase information corresponding to the 40 Hz signal measured by our wearable EEG system. This phase information was then used to calculate the PLI. The results of this study indicate that the phase information associated with the 40 Hz ASSR was particularly robust against artifacts induced by walking and eye movements. The artifact caused by walking mainly occurs when each foot lands on the floor. The artifact caused by eye movements occurs only when the participants move their eyes. Therefore, the artifacts are transient, although the amplitudes of the artifacts are extremely high compared with that of EEG. For the PLI calculation, we only used phase information associated with 40 Hz. This corresponds to the normalization of amplitude information. Thus, the phase information is robust against the artifact caused by walking and eye movements. The results of this study suggest that the PLI can be a good indicator to estimate the cognitive workload during walking with a limited EEG recording. Previous studies have measured EEG during walking by using a spatial filter. We reduced the artifacts in the time domain rather than in the spatial domain. Thus, we succeeded in estimating the workload from EEG data recorded with a few electrodes. Furthermore, several studies have reported measuring the workload during walking using mobile EEG systems (De Sanctis et al., 2014; Marcar et al., 2014; Beurskens et al., 2016). These researchers investigated the cognitive and motor workload in dual tasks in which participants performed another motor and cognitive task in addition to walking. However, they used different physical stimuli or different experimental tasks to modulate the workload. Therefore, it is not clear whether the workload was caused by the change in the cognitive and motor workload itself or the change in the workload due to different experimental modality. To avoid this problem, we compared the workload by using an N-back task, in which the visual stimuli were same throughout the experiment with constant walking speed.

In this study, as we did not perform any noise reduction processing, it is quite likely that the PLI was affected by the artifact to some extent. Previous studies that measured EEG during walking used ICA and canonical correlation analysis (CCA) for noise reduction (Bulea et al., 2014; Kline et al., 2014; Lau et al., 2014; Lin et al., 2014; Wagner et al., 2014). The advantage of the PLI analysis is that it can be used for EEG data collected from few electrodes. However, if we had recorded EEG data from many electrodes, we could have also used ICA and CCA for noise reduction. Previous studies have succeeded in extracting steady-state responses from multiple channels (Zhu et al., 2013; Bharadwaj and Shinn-Cunningham, 2014). Indeed, such methods could enable the estimation of brain workload with higher precision.

In the present study, we estimated human workload while participants walked in a naturalistic environment. Participants were outfitted with a custom wearable EEG system. Our results suggest that the ASSR can be used to estimate workload in an ecologically valid environment when participants are engaged in a variety of tasks.

## Author Contributions

YN designed the experiment(s), ST and AM created the custom-designed headgear and YY conducted the experiment(s), wrote the main manuscript text and prepared all the figures. All authors reviewed the manuscript.

## Conflict of Interest Statement

The authors declare that the research was conducted in the absence of any commercial or financial relationships that could be construed as a potential conflict of interest.
